# Skeletal muscle atrogene expression and insulin resistance in a rat model of polytrauma

**DOI:** 10.14814/phy2.12659

**Published:** 2016-01-27

**Authors:** Robert M. Akscyn, John L. Franklin, Tatyana A. Gavrikova, Joseph L. Messina

**Affiliations:** ^1^ Department of PathologyDivision of Molecular and Cellular PathologyUniversity of Alabama at BirminghamBirminghamAlabama; ^2^ Vetrans Affairs Medical CenterBirminghamAlabama

**Keywords:** Burn, cecal ligation and puncture, insulin signaling, proinflammatory cytokines, skeletal muscle atrophy, TRIB3

## Abstract

Polytrauma is a combination of injuries to more than one body part or organ system. Polytrauma is common in warfare, and in automobile and industrial accidents. The combination of injuries can include burn, fracture, hemorrhage, and trauma to the extremities or specific organ systems. Resistance to anabolic hormones, loss of muscle mass, and metabolic dysfunction can occur following injury. To investigate the effects of combined injuries, we have developed a highly reproducible rodent model of polytrauma. This model combines burn injury, soft tissue trauma, and penetrating injury to the gastrointestinal (GI) tract. Adult, male Sprague–Dawley rats were anesthetized with pentobarbital and subjected to a 15–20% total body surface area scald burn, or laparotomy and a single puncture of the cecum with a G30 needle, or the combination of both injuries (polytrauma). In the current studies, the inflammatory response to polytrauma was examined in skeletal muscle. Changes in skeletal muscle mRNA levels of the proinflammatory cytokines TNF‐α, IL‐1β, and IL‐6 were observed following single injuries and polytrauma. Increased expression of the E3 ubiquitin ligases Atrogin‐1/FBX032 and TRIM63/MuRF‐1 were measured following injury, as was skeletal muscle insulin resistance, as evidenced by decreased insulin‐inducible insulin receptor (IR) and AKT/PKB (Protein Kinase B) phosphorylation. Changes in the abundance of IR and insulin receptor substrate‐1 (IRS‐1) were observed at the protein and mRNA levels. Additionally, increased TRIB3 mRNA levels were observed 24 h following polytrauma, the same time when insulin resistance was observed. This may suggest a role for TRIB3 in the development of acute insulin resistance following injury.

## Introduction

Polytrauma is most simply defined as multiple, simultaneous injuries to more than one body part or organ system (Butcher and Balogh 2009). Polytrauma is common in modern warfare; in recent military conflicts, significant burn injury with concomitant penetrating injury were common in closed‐space explosion events, such as within vehicles (Ramasamy et al. 2009, 2011; Dismounted Complex Blast Injury Task Force, 2011). Civilians are also susceptible to polytrauma through automobile and industrial accidents (Santaniello et al. 2004; Atiyeh et al. 2007; Owens et al. 2008). Polytrauma can include burn injury, fracture, hemorrhage, trauma to the extremities, or penetrating trauma to the gastrointestinal (GI) tract. These injuries are highly survivable when occurring individually. However, when they occur in combination a complex pathophysiology is observed, the prognosis is worsened, and mortality increased (Pfeifer et al. 2009; Shere‐Wolfe et al. 2012). Advances in battlefield medicine and emergency response strategies have increased initial survival following polytrauma, but later complications (multiple organ failure, metabolic dysfunction, severe infection) result in high mortality (Keel and Trentz 2005; Pfeifer et al. 2009; Sritharan and Thompson 2009; Shere‐Wolfe et al. 2012). The effects of polytrauma are tissue specific, but poorly understood due to the lack of clinically relevant models. However, the responses of the immune system combined with those of multiple organ systems contribute to the systemic pathophysiology (Neunaber et al. 2011).

To investigate the effects of polytrauma, we have developed a rodent model of simultaneous burn, soft tissue trauma, and penetrating injury of the bowel. This model combined burn injury with cecal ligation and puncture (CLP). Cecal ligation and puncture has long been used to study sepsis and peritonitis, and can be viewed as polytrauma itself, as the laparotomy produces soft tissue trauma and the penetrating damage to the GI tract as a second injury. However, for the purposes of these studies a minor CLP injury has been utilized to mimic a penetrating, lower abdominal injury that perforates the bowel as often occurs in blast injuries, or in automobile or industrial accidents (Santaniello et al. 2004; Atiyeh et al. 2007; Owens et al. 2008). Importantly, both the burn, 15–20% total body surface area (TBSA), and the CLP, G30 needle with a single puncture of the cecum, used in this study are much less severe than those typically utilized in burn and sepsis studies (Herndon et al. 1978; Wichterman et al. 1980; Claassen et al. 2014; Diao et al. 2014; Nardi et al. 2014; Peng et al. 2014; Pidcoke et al. 2014; Tsao et al. 2014). The crux of our model is the simultaneous timing of the injuries, which is distinct from “double hit” models, where CLP is performed one or more days after burn injury to model the infection often observed in burn patients (Orman et al. 2012; Mattick et al. 2013).

In this study, we sought to investigate the effects of multiple combined minor injuries, polytrauma, on the inflammatory response in skeletal muscle, in particular the local expression of proinflammatory cytokines in skeletal muscle. We hypothesized that polytrauma would result in increased proinflammatory cytokine production, including IL‐1β and IL‐6. We also hypothesized that polytrauma would result in skeletal muscle insulin resistance. Thus, we investigated skeletal muscle insulin signaling, which revealed changes to insulin receptor and insulin receptor substrate‐1 protein, and messenger RNA levels following polytrauma. Skeletal muscle insulin resistance was measurable as reduced insulin activation of the insulin receptor and AKT/PKB (Protein Kinase B), which may involve changes in Tribbles homolog 3 (TRIB3) gene expression and/or increased proinflammatory cytokine mRNA levels in skeletal muscle following polytrauma. Additionally, we hypothesized that due to increased proinflammatory cytokine production and the development of insulin resistance, polytrauma would result in enhanced muscle proteolysis, including induction of ubiquitin E3 ligases involved in the ubiquitin‐proteasome degradation pathway in skeletal muscle.

## Materials and Methods

### Animal model of polytrauma (burn/CLP)

All animal procedures were carried out in accordance with the guidelines set forth in the Animal Welfare Act and the Guide for the Care and Use of Laboratory Animals by the National Institutes of Health (Bethesda, Maryland). The experimental protocol was approved by the Institutional Animal Care and Use Committee at the University of Alabama at Birmingham. Specific pathogen‐free, male Sprague–Dawley rats from Harlan Laboratories, 12 weeks of age, with an average weight of 290 g were used throughout this study. Prior to experiments, rats were acclimatized after arrival for at least 1 week in a 12‐h light/dark cycle and room temperature at 22.2 ± 1.0°C. The humidity of the animal facility was between 22% and 45% during these studies. Rats had free access to water and food (NIH‐31 Open Formula Diet from Harlan Laboratories, catalog #7917). Rats were group‐housed, three rats per cage, prior to the procedures. Following injury, rats were individually housed until the time of euthanasia. Animals were not fasted prior to manipulation. Rats were anesthetized by brief inhalation of 2% isoflurane, followed by injection of sodium pentobarbital (60 mg/kg i.p.).

### Burn injury

Animals were placed in a supine position in a plastic template, which exposed the dorsum. A full‐thickness skin scald burn was inflicted by immersing the back of the animal in 95°C water for 10 sec. To calculate the percentage of total burn surface area (TBSA), the burn area was immediately traced, measured and TBSA of each burned animal was calculated using Meeh's formula (Total Body Surface Area = kW^0.667^) with a Meeh constant of 9.46 (Gilpin 1996). Based on these measurements, an approximate 15–20% TBSA burn was produced. For sham burn, rats were submerged in 37°C water for 10 sec. Rats were quickly dried and while still under pentobarbital anesthesia, cecal ligation and puncture (CLP) or sham/CLP was performed.

### Cecal ligation and puncture

Following a 2‐cm ventral midline laparotomy, the cecum was exposed and ligated just distal to the ileocecal valve to avoid intestinal obstruction. A G30 needle was used to puncture the cecum once, not a “through and through” puncture of both sides of the cecum and a small amount of the bowel content was extruded to insure patency of the opening. The cecum was returned to the abdominal cavity and the incision closed in layers. Sham‐operated animals underwent the same surgical procedure with the cecum being exposed except the cecum was neither ligated nor punctured. All animals received resuscitation fluid (0.9% sterile saline, 30 mL/kg, warmed to 37°C) via a subcutaneous injection in the nape of the neck with care not to disturb the burn area. Rats were returned to the cages and thermal support provided (heating pad set to 37°C) until consciousness and mobility were regained. Water was provided ad libitum. Chow was not provided to animals in the 6‐h groups. Chow was provided to animals in the 24‐h groups until a fasting period of 20 h prior to euthanasia. At endpoints, rats were anesthetized with 2% isoflurane inhalation, the laparotomy was reopened and insulin (5 U/kg) dissolved in saline or an equal volume of saline alone was injected into the inferior vena cava. Triceps were harvested 2 min after insulin injection and immediately snap frozen in liquid nitrogen. To ensure euthanasia, the diaphragm was cut and the heart excised. No animals in any treatment group expired prior to the planned euthanasia timepoint.

### Experimental groups

This study used four treatment groups. To account for the effects of anesthesia and surgical manipulation a double‐sham group (sham/sham), sham burn with sham CLP served as the control group. To discern the effects of the single injuries, burn injury alone with a sham CLP (burn/sham) or CLP injury alone with a sham burn (sham/CLP) was performed. To investigate the effects of polytrauma, a double injury, burn plus CLP (burn/CLP), was performed.

### Western blotting and densitometry

Triceps tissue (0.2 g) from each animal was homogenized in lysis buffer as described previously (Xu et al. 2008; Li et al. 2009a). The tissue lysates were centrifuged, and the supernatants were stored at −80°C until use. Lysate protein concentrations were assayed by BCA assay following the manufacturer's instructions (Pierce, Rockford, Ill). The lysates (15 *μ*g/lane) were subjected to sodium dodecyl sulfate polyacrylamide gel electrophoresis and transferred to nitrocellulose membranes. Membranes were immunoblotted with the following primary antibodies: anti–total insulin receptor (Santa Cruz Biotechnology, Santa Cruz, CA), anti–total insulin receptor substrate‐1 (Millipore, Billerica, MA), anti‐PY1150/1151‐insulin receptor, anti‐PS473‐AKT, and anti‐GAPDH (Cell Signaling, Danvers, Massachusetts). Membranes were not stripped and reprobed for GAPDH or any other protein, but the blot was cut using molecular weight markers as a guide, and the top and bottom halves of the blots were probed with different specific antibodies. Entire blots are presented in Figure 3; subsequent Western data is presented as representative individual lanes captured from the same film.

Enhanced chemiluminescence images of immunoblots were obtained on CL‐X Posure Film (Thermo Scientific, Rockford, Illinois). Films were scanned using a Hewlett‐Packard C5280 scanner, and relative band intensities were determined using Zero D‐Scan (Scanalytics Corp, Fairfax, VA) (Xu et al. 2008; Li et al. 2009a).

### Total RNA extraction

Triceps were frozen in liquid nitrogen until extraction of total cellular RNA using the Aurum Total RNA Fatty and Fibrous Kit (Bio‐Rad, Hercules, CA) and treated with DNase I on the column according to the manufacturer's recommendations. Briefly, 100 mg of triceps tissue was ground into a fine powder under liquid nitrogen and homogenized in PureZOL. Following centrifugation, ethanol was added to the supernatant and the lysate transferred to an RNA‐binding column. After a low‐stringency wash, DNase I treatment, a high‐stringency wash and an additional low‐stringency wash, the RNA was eluted. RNA concentration and quality was determined by spectrophotometry at 260/280 nm and samples were stored at −80°C.

### Quantitative real‐time PCR

RNA samples were used as a template for the reverse transcriptase reaction to generate cDNAs using the High Capacity Reverse Transcriptase Synthesis kit (Applied Biosystems, Foster City, CA) according to the manufacturer's protocol. RNA (1 *μ*g) was reverse transcribed in 20 *μ*L reaction volume containing Reaction Mix with a blend of oligo (dT) and random hexamer primers, and reverse transcriptase. cDNA samples were diluted 1:10 and 1 *μ*L of diluted cDNA was used in each 25 *μ*L real‐time PCR reaction, using the iQ Supermix (Bio‐Rad) with the iQ5 Real Time PCR Detection System (Bio‐Rad). The mRNA expression level of each gene was quantified using the oligonucleotide primers specially designed and optimized for this study using PrimerQuest (Integrated DNA Technologies, Coralville, IA). Each sample was run in triplicate, and the mean threshold cycle value of target genes was normalized to the expression of 18S rRNA in the corresponding sample. Fold expression of genes was calculated using the comparative C_T_ (ΔΔC_T_) method (fold expression = 2^−ΔC^_T_^(target) − ΔC^_T_^(18S)^). The following primer sequences were used; insulin receptor Forward (5′‐TGT GGC CTG TCG CAA CTT CTA TCT‐3′), Reverse (5′‐AGT GAA GGT CTT GGC AGA AGC TGA‐3′); insulin receptor substrate‐1 Forward (5′‐ACC GAC GCA TGG TCT ATG TTG CTA‐3′), Reverse (5′‐AAC CTG GCC TGG TAC CCA TTA CAT‐3′); *TNF‐α* Forward (5′‐CGT AGC CCA CGT CGT AGC‐3′), Reverse (5′‐GTC CCT TGA AGA GAA CCT GGG AGT‐3′); *IL‐1β* Forward (5′‐AAG AGC TTC AGG GCA GTGTCA‐3′), Reverse (5′‐TGG GAA CAT CAC ACA CTA GCA GGT‐3′); *IL‐6* Forward (5′‐AAC TCC ATC TGC CCT TCA GGA ACA‐3′) Reverse (5′‐AAG GCA GTG GCT GTC AAC AAC ATC‐3′); *TRIB3* Forward (5′‐GAG TAC TGG TGT CTC AGC TTT C‐3′), Reverse (5′‐GCA CAA TGG CTG TTT CTT CC‐3′).

### Statistical analysis

Data are presented as mean ± SEM. Data were analyzed using the InStat statistical program (GraphPad Software, Inc., San Diego, California). Differences between groups were determined using one‐way ANOVA (Tukey post‐test) or Student's *t‐*test (two‐tailed, unpaired, Welch‐corrected). Comparisons were made at a single timepoint and not between timepoints. Unless otherwise noted, significant differences are denoted as a = *P* < 0.05 versus sham/sham, b = *P* < 0.05 versus burn/sham, and c = *P* < 0.05 versus sham/CLP.

## Results

### Proinflammatory cytokine mRNA levels in triceps

**Figure 1:**
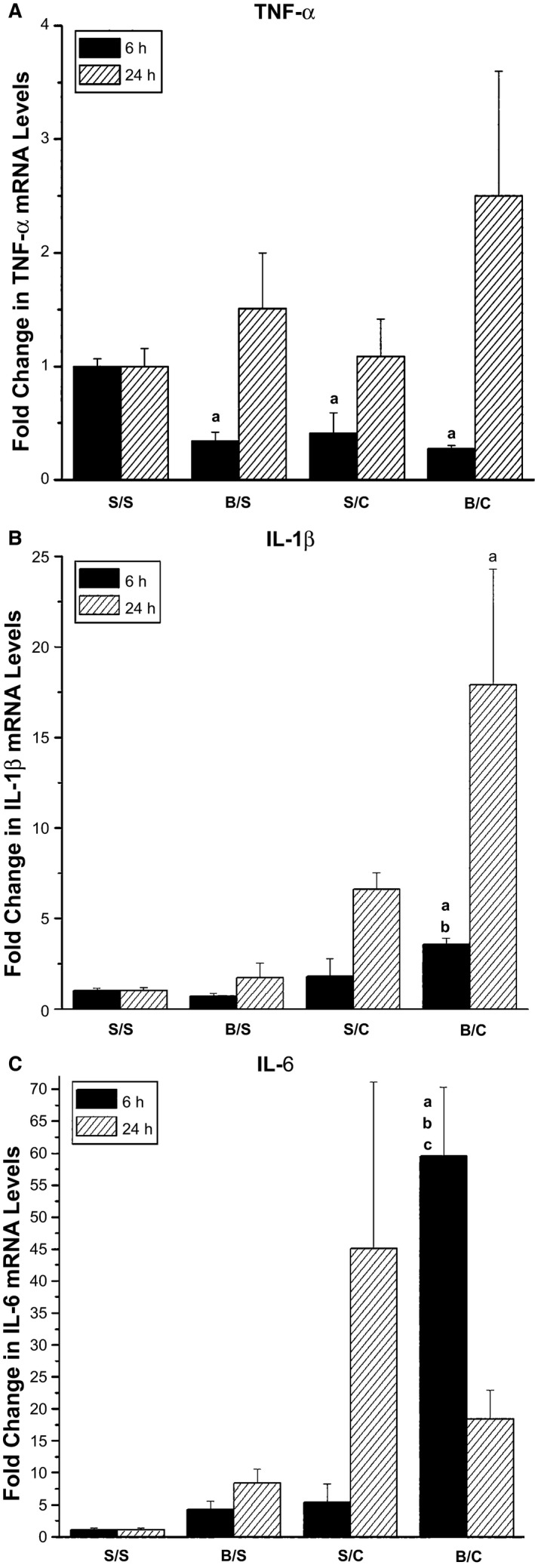
Increased proinflammatory cytokine mRNA levels in triceps at 6 h and 24 h following injury. Rats were subjected to double sham (S/S), burn injury alone (B/S), cecal ligation and puncture alone (S/C), or the combination of burn and cecal ligation and puncture (B/C). At 6 h and 24 h, rats were euthanized and triceps harvested. (A) Quantitative real‐time PCR was used to analyze TNF‐α mRNA levels (*N* = 3 for S/S, B/S, S/C and 6 for B/C at both 6 h and 24 h). (B) Quantitative real‐time PCR was used to analyze IL‐1β mRNA levels (*N* = 3 for S/S, B/S, S/C and 6 for B/C at 6 h, *N* = 4 for S/S and 6 for B/S, S/C and B/C at 24 h). (C) Quantitative real‐time PCR was used to analyze IL‐6 mRNA levels (*N* = 3 for S/S, B/S, S/C and 6 for B/C at 6 h, *N* = 4 for S/S and 6 for B/S, S/C and B/C at 24 h). The data are presented as the mean ± SEM and values were normalized to the time‐matched S/S group. Statistical significance was assessed with a one‐way ANOVA with a Tukey post‐test. The threshold of significance was set at *P* < 0.05. Significance is denoted as, a = significant versus sham/sham, b = significant versus burn/sham, c = significant versus sham/CLP. Additional statistical analysis with Student's *t*‐tests (two‐tailed, unpaired, Welch‐corrected) was also performed and demonstrated the increases in IL‐1β in the sham/CLP group at 24 h were significant versus sham/sham and burn/sham, and the increases in the burn/CLP group were significant versus all other groups. Further, the increases in IL‐6 observed in the burn/sham and burn/CLP groups at 24 h were significant versus sham/sham by *t*‐test. However, these statistics are not added to the figure itself which presents statistics performed by ANOVA analysis.

Increases in proinflammatory cytokines occur following burn and CLP. To determine the effects of combined injury, polytrauma, on proinflammatory cytokine production in skeletal muscle mRNA levels of TNF‐α, IL‐1β, and IL‐6 were measured at 6 and 24 h following injury. Unexpectedly, at the 6‐h timepoint, skeletal muscle TNF‐α message levels were significantly decreased by both single injuries and burn/CLP versus sham/sham (Fig. 1A). At 24 h, there were no significant differences in TNF‐α message levels among groups (Fig. 1A). Thus, skeletal muscle may not be a major source of TNF‐α in the single or combined injuries.

Interleukin‐1β message levels were modestly increased in response to polytrauma (burn/CLP) at 6 h (Fig. 1B). At 24 h, IL‐1β mRNA levels were significantly increased in the burn/CLP group versus sham/sham (Fig. 1B).

Six hours following injury IL‐6 mRNA levels were significantly increased, approximately 60‐fold, only in the polytrauma (burn/CLP) group (Fig. 1C). At 24 h, animal‐to‐animal variability in the sham/CLP group precluded increases from reaching significance (Fig. 1C).

### E3 ubiquitin ligase mRNA levels in triceps following injury

**Figure 2:**
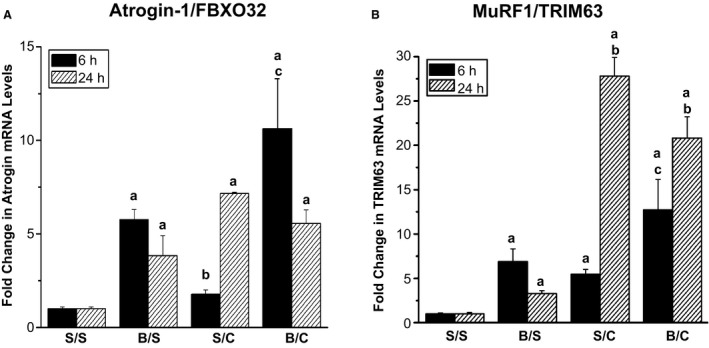
Increased atrogene mRNA levels in triceps at 6 h and 24 h following injury. Rats were subjected to double sham (S/S), burn injury alone (B/S), cecal ligation and puncture alone (S/C), or the combination of burn and cecal ligation and puncture (B/C). At 6 h and 24 h, rats were euthanized and triceps harvested. (A) Quantitative real‐time PCR was used to analyze Atrogin‐1/FBXO32 mRNA levels (*N* = 3 for S/S, B/S, S/C and 6 for B/C at both 6 h and 24 h). (B) Quantitative real‐time PCR was used to analyze MuRF‐1/TRIM63 mRNA levels (*N* = 3 for S/S, B/S, S/C and 6 for B/C at both 6 h and 24 h). The data are presented as the mean ± SEM and values were normalized to the time‐matched S/S group. Statistical significance was assessed with a one‐way ANOVA with a Tukey post‐test. The threshold of significance was set at *P* < 0.05. Significance is denoted as, a = significant versus sham/sham, b = significant versus burn/sham, c = significant versus sham/CLP.

Muscle atrophy can occur following injury and proinflammatory cytokines induce muscle wasting (Tsujinaka et al. 1996; Bodine et al. 2001; Li et al. 2005, 2009b; Frost et al. 2007; Lang et al. 2007; Bonaldo and Sandri 2013; Castillero et al. 2013; Merritt et al. 2013; Tang et al. 2014; Ma et al. 2015; Yuan et al. 2015). To study the effects of polytrauma on muscle atrophy, mRNA levels of Atrogin‐1 (FBXO32) and MuRF‐1 (TRIM63) were examined. Atrogin‐1 mRNA levels increased 6 h following sham/burn and burn/CLP, but there was no significant change in the sham/CLP group compared to the sham/sham group (Fig. 2A). At the 24‐h timepoint, burn or CLP alone, or the combination of injuries all resulted in increased Atrogin‐1 mRNA levels (Fig. 2A). MURF‐1 mRNA levels were increased at 6 h by either single injury alone or the combination of burn/CLP (Fig. 2B). At 24 h, MURF‐1 levels remained significantly elevated in the burn/sham group (Fig. 2B). However, CLP resulted in a dramatic increase in MURF‐1 mRNA levels as both the sham/CLP and burn/CLP groups resulted in 20‐ and 30‐fold increases (Fig. 2B).

### Insulin receptor protein and mRNA levels in triceps following polytrauma

**Figure 3:**
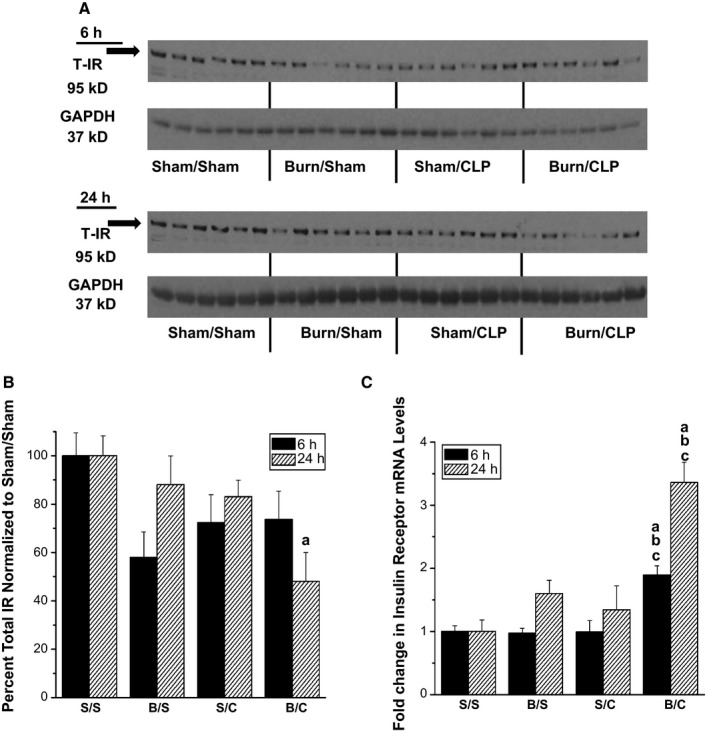
Altered insulin receptor protein and mRNA levels in triceps following injury. Rats were subjected to double sham (S/S), burn injury alone (B/S), cecal ligation and puncture alone (S/C), or the combination of burn and cecal ligation and puncture (B/C). Six hours and 24 h after injury rats were euthanized and triceps were harvested. (A,B) Western blot analysis with anti–total insulin receptor specific antibody. The levels of total insulin receptor were measured and quantified by scanning densitometry of the autoradiographs. GAPDH was used to demonstrate even loading. Total insulin receptor was not corrected to GAPDH (*N* = 6 for all groups at both 6 h and 24 h). (C) Quantitative real‐time PCR was used to analyze insulin receptor mRNA levels (*N* = 3 for S/S, B/S, S/C and 6 for B/C at 6 h, *N* = 5 for S/S and 6 for B/S, S/C and B/C at 24 h). The data are presented as the mean ± SEM and the values normalized to the sham/sham group. Statistical significance was assessed with a one‐way ANOVA with a Tukey post‐test. The threshold of significance was set at *P* < 0.05. Significance is denoted as, a = significant versus sham/sham, b = significant versus burn/sham, c = significant versus sham/CLP.

To understand the effects of polytrauma on skeletal muscle insulin signaling, the amount of insulin receptor (IR) protein was first examined at 6 h and 24 h following injury. At the 6‐h timepoint, there were no significant differences for IR protein present between groups (Fig. 3A,B). At 24 h postinjury, there was a reduction in the amount of IR protein in the burn/CLP group, which was significantly less than sham/sham (Fig. 3A).

To determine if these changes in IR protein levels resulted from altered gene expression, quantitative real‐time PCR was used to measure mRNA levels. At the 6‐h timepoint, there was a 2‐fold increase in IR mRNA levels in the polytrauma (burn/CLP) group, which was significantly higher than sham/sham or either single injury (Fig. 3C). Twenty‐four hours following injury, the burn/CLP group again had significantly higher IR mRNA levels versus all other groups.

### Polytrauma‐induced insulin resistance in triceps

**Figure 4:**
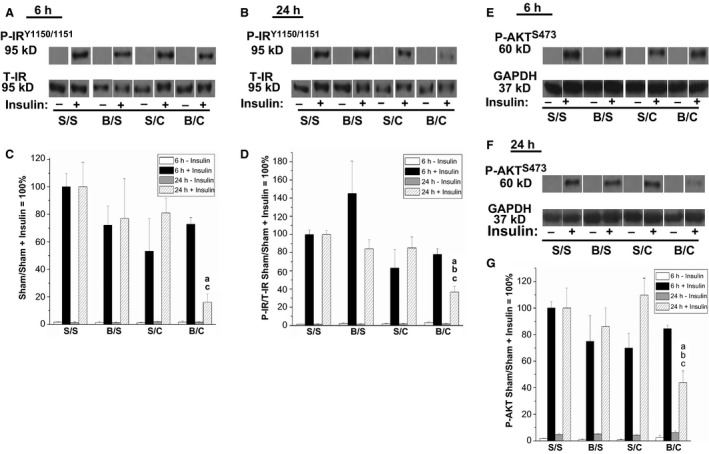
Skeletal muscle insulin resistance following polytrauma. Rats were subjected to double sham (S/S), burn injury alone (B/S), cecal ligation and puncture alone (S/C), or the combination of burn and cecal ligation and puncture (B/C). Six hours and 24 h after injury rats were anesthetized and either saline (−) or 5 U/kg insulin (+) was injected into the vena cava. The triceps were removed 2 min later and subjected to Western blot analysis. (A,B,C,D) Western blot analysis with specific anti‐PY1150/1151‐IR antibody. The levels of PY1150/1151‐IR were measured and quantified by scanning densitometry of the autoradiographs. The PY1150/1151‐IR signal was quantified with and without correction for total‐IR abundance: the left panel bar graph (C) is without total‐IR correction and the right panel bar graph (D) is following correction for total‐IR abundance (*N* = 3 minus and 3 plus insulin for all groups at both 6 h and 24 h). (E,F,G) Western blot analysis with specific anti‐PS473‐AKT antibody. The levels of PS473‐AKT were measured and quantified by scanning densitometry of the autoradiographs. GAPDH was used to demonstrate even loading. PS473‐AKT was not corrected to GAPDH (*N* = 3 minus and 3 plus insulin for all groups at both 6 h and 24 h). In A, B, E, and F representative lanes from the scanned image of a single film are shown. The data are presented as the mean ± SEM and the sham/sham plus insulin group was set equal to 100% at each timepoint. Statistical significance was assessed with a one‐way ANOVA with a Tukey post‐test. The threshold of significance was set at *P* < 0.05. Significance is denoted as, a = significant versus sham/sham, b = significant versus burn/sham, c = significant versus sham/CLP.

Skeletal muscle insulin signaling was assessed with Western blotting for tyrosine phosphorylation of the insulin receptor (PY1150/51‐IR) in response to exogenous insulin treatment. Insulin induction of PY1150/51‐IR was assessed in two ways, based solely on the PY1150/51‐IR signal and, since there were decreased total‐IR levels in the polytrauma group at 24 h, the PY1150/51‐IR signal was corrected for the total amount of IR protein present. Both methods of analysis are shown in (Fig. 4C,D). At the 6‐h timepoint, there were no significant differences observed in IR phosphorylation between groups (Fig. 4C,D). This data demonstrates that at 6‐h postinjury the same amount of insulin receptors are phosphorylated in response to exogenous insulin treatment in all groups. Examination of phosphorylated IR at the 24‐h timepoint without correcting for the amount of total‐IR, revealed a decrease in the polytrauma (burn/CLP) group that was significant compared to the sham/sham and sham/CLP groups (Fig. 4C). When the changes in cellular levels of total‐IR are accounted for, the significant reduction in the PY1150/51‐IR signal remained in the polytrauma (burn/CLP) group compared to the sham/sham and both single injury groups (Fig. 4D). Therefore, at 24 h following polytrauma skeletal muscle insulin resistance, as measured by insulin‐induced IR tyrosine phosphorylation, was evident by a decreased ability of the insulin receptors present to become phosphorylated/activated in response to insulin treatment. Even when accounting for the decreased amount of total‐IR present, the remaining insulin receptors were less able to be tyrosine phosphorylated following insulin treatment.

Insulin signaling downstream of the receptor was examined with Western blotting for phosphorylated AKT (S473‐AKT). Six hours following injury, there were no significant differences in the amount of AKT phosphorylated in response to exogenous insulin treatment (Fig. 4E,G). However, at the 24‐h timepoint, PS473‐AKT was significantly decreased in the polytrauma (burn/CLP) group versus sham/sham and both single injury groups (Fig. 4F,G). These results suggest that signal amplification was insufficient to compensate for the deficit of insulin‐induced signaling at the level of the insulin receptor and that insulin resistance is measurable in skeletal muscle at multiple steps in the insulin‐signaling pathway 24 h following polytrauma.

### Injury‐induced changes to insulin receptor substrate‐1 and TRIB3 in triceps

**Figure 5:**
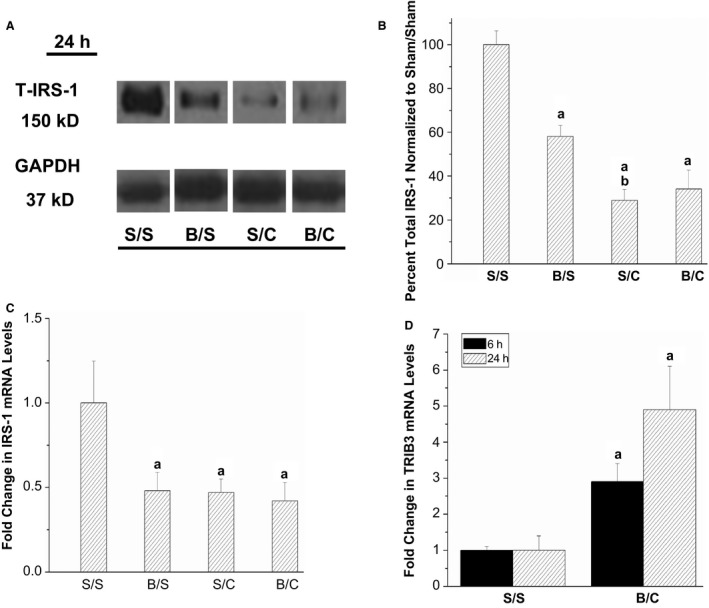
Changes to protein and mRNA levels of insulin receptor substrate‐1 and TRIB3 24 h following injury. Rats were subjected to double sham (S/S), burn injury alone (B/S), cecal ligation and puncture alone (S/C), or the combination of burn and cecal ligation and puncture (B/C). At 24 h, rats were euthanized and triceps harvested. (A,B) Western blot analysis with anti–total insulin receptor‐1‐specific antibody. The levels of total‐IRS‐1 were measured and quantified by scanning densitometry of the autoradiographs. GAPDH was used to demonstrate even loading. Total IRS‐1 was not corrected to GAPDH (*N* = 6 for all groups). Representative lanes from the scanned image of a single film are shown. (C) Quantitative real‐time PCR was used to analyze IRS‐1 mRNA levels (*N* = 5 for S/S and *N* = 6 for B/S, S/C and B/C). (D) Quantitative real‐time PCR was used to analyze TRIB3 mRNA levels at 6 h and 24 h (*N* = 3 for S/S and 6 for B/C at both 6 h and 24 h). The data are presented as the mean ± SEM and the values were normalized to the time‐matched S/S group. Statistical significance was assessed with a one‐way ANOVA with a Tukey post‐test in A and B. The threshold of significance was set at *P* < 0.05. Significance is denoted as, a = significant versus sham/sham, b = significant versus burn/sham, c = significant versus sham/CLP. Student's *t*‐test (two‐tailed, unpaired, Welch‐corrected) was used for statistical analysis in C. The threshold of significance was set at *P* < 0.05 and significance is denoted as a = significant versus sham/sham.

To further investigate the effects of polytrauma on the insulin‐signaling pathway, protein and mRNA levels of insulin receptor substrate‐1 (IRS‐1) were measured. At the 24‐h timepoint, IRS‐1 protein levels were decreased by both single injuries and burn/CLP (Fig. 5A,B).

Insulin receptor substrate‐1 mRNA levels were examined to determine if the observed decreases in IRS‐1 protein were due to decreased gene expression/transcript stability. Twenty‐four hours following injury, IRS‐1 mRNA levels were significantly reduced by both single injuries and burn/CLP (Fig. 5C).

We and others have demonstrated a role of Tribbles homolog 3 (TRIB3) in skeletal muscle insulin resistance (Liu et al. 2010; Matos et al. 2010). Thus, we tested whether TRIB3 mRNA might be increased, suggesting it could contribute to the skeletal muscle insulin resistance observed following polytrauma. TRIB3 mRNA levels were measured at 6 h and 24 h following injury. At 6 h, burn/CLP resulted in a modest, but significant increase in TRIB3 mRNA levels versus sham/sham (Fig. 5D). At 24 h, TRIB3 mRNA levels remained significantly elevated by burn/CLP. This data suggests increasing TRIB3 mRNA levels following burn/CLP may play a role in the development of polytrauma‐specific insulin resistance.

## Discussion

Multiple, simultaneous injuries are common on the battlefield as well as in civilian accidents. Projections suggest polytrauma injury patterns will increase along with the increase in global industrialization (Lecky et al. 2010). Individually, burn injury and CLP are known to increase expression of proinflammatory cytokines in skeletal muscle in both human and animal studies (Lang and Frost 2007; Sheriff et al. 2009; Merritt et al. 2013; Steiner and Lang 2015). In this study, a minimal burn injury or minor CLP resulted in increased IL‐1β and IL‐6 mRNA levels in skeletal muscle, but polytrauma resulted in greater increases. Cumulatively, this data demonstrates a greater inflammatory response in triceps following polytrauma, which may contribute to systemic pathology following combined injuries. This suggests a synergy of the combined injuries resulting in a hyperinflammatory response to polytrauma in skeletal muscle. The lack of an increase in skeletal muscle TNF‐α mRNA levels following injury was unexpected. Increased skeletal muscle TNF‐α mRNA levels have been found 24 h following burn injury and CLP in rats (Lang and Frost 2007; Quintana et al. 2015). It is possible the two endpoints, 6 h and 24 h, examined in this study missed a TNF‐α peak or that the less severe burn injury, and the less severe CLP used were insufficient to elicit an increase in TNF‐α mRNA levels.

The signaling cascades and transcriptional responses to proinflammatory cytokines elicit expression of genes involved with the ubiquitin‐proteasome protein degradation pathway leading to muscle atrophy and has been demonstrated in both in vivo and in vitro studies (Tsujinaka et al. 1996; Bodine et al. 2001; Li et al. 2005, 2009b). Increased expression of the ubiquitin E3 ligases, Atrogin‐1/FBXO32 and MURF‐1/TRIM63 have been demonstrated in numerous pathological conditions leading to muscle atrophy (Frost et al. 2007; Lang et al. 2007; Bonaldo and Sandri 2013; Castillero et al. 2013; Merritt et al. 2013; Tang et al. 2014; Ma et al. 2015; Yuan et al. 2015). In the current studies, we observed expression of Atrogin‐1/FBXO32 to be more strongly burn‐driven at 6 h following injury, with the effects CLP becoming more evident at the 24‐h timepoint. The expression of MuRF‐1/TRIM63 was induced at 6 h by both burn injury and CLP; at the 24‐h timepoint, the increase in MuRF‐1 mRNA levels was again more highly CLP dependent. The induction of these atrogenes at 6 h is earlier than what is typically observed. Further, the fold‐increase of these atrogenes, MuRF‐1 in particular, is much higher than what is typically reported in injury and chronic disuse/disease models. These findings suggest a rapid priming of the ubiquitin‐proteasomal protein degradation pathway and the potential for loss of muscle mass, particularly following combined injury (polytrauma).

Hyperglycemia and insulin resistance following injury is associated with increased morbidity and mortality. Insulin has been demonstrated to have potent anti‐inflammatory effects and protect against organ damage in critically ill patients (Hansen et al. 2003; Jeschke et al. 2004; Xu et al. 2005; Leffler et al. 2007; Bortoff et al. 2010). Additionally, increases in skeletal muscle atrogenes is known to occur under conditions of insulin resistance. Understanding the causative factors of injury‐induced insulin resistance and the tissue specific differences is crucial to providing the best treatment and care for patients. A goal of this work was to determine if insulin signaling was impaired in skeletal muscle by our model of polytrauma. The data demonstrated altered protein and mRNA levels of insulin receptor and insulin receptor substrate‐1 following injury. Cumulatively, the results demonstrated that IR protein levels decreased in triceps following polytrauma and that decreased mRNA levels were not responsible for the reduced protein levels. Instead, increased IR mRNA may suggest an attempted compensation for the decreased IR protein levels following polytrauma.

Skeletal muscle insulin resistance was observed 24 h following polytrauma and was specific to polytrauma. We have previously reported acute insulin resistance in skeletal muscle in a model of trauma and hemorrhage (Thompson et al. 2008; Zhai and Messina 2009). Studies from other groups have also demonstrated skeletal muscle insulin resistance following a single injury more severe than those in this work (Nunes et al. 2001; Sugita et al. 2005; Calisto et al. 2012; Nakazawa et al. 2015), but this was not universal (Matsuda et al. 2009). A possible explanation for these divergent results may be the different routes of insulin treatment and timing of measurement of insulin signaling. In this work, decreased IR protein levels were observed following polytrauma at 24 h. None of the previously mentioned studies (Nunes et al. 2001; Sugita et al. 2005; Matsuda et al. 2009; Calisto et al. 2012; Nakazawa et al. 2015) demonstrated decreased IR protein levels following injury. To our knowledge, skeletal muscle IR mRNA levels following injury have not been reported. Reduction in IRS‐1 protein levels in skeletal muscle has been documented in sepsis and following burn injury, and is primarily mediated through IRS‐1 serine phosphorylation and subsequent proteosomal degradation (Nunes et al. 2001; Barreiro et al. 2004; Zhang et al. 2005; Lu et al. 2013). Serine phosphorylation of IRS‐1 was not observed in our model (data not shown), although decreased IRS‐1 mRNA levels were evident and are a possible explanation for the reduction in IRS‐1 protein levels.

However, taken together, these results suggest the reduced IRS‐1 protein (and mRNA) levels observed in burn/sham and sham/CLP were not sufficient to decrease downstream insulin signaling, because there was no decrease, as measured by changes in P‐AKT. Thus, the reduced IRS‐1 protein levels observed in skeletal muscle following polytrauma may not be a major contributor to the polytrauma‐specific insulin resistance observed at 24 h. But in the context of polytrauma‐driven decreased insulin receptor phosphorylation/activation, the decrease in IRS‐1 protein may contribute to decreased downstream insulin signaling.

In addition to changes in insulin‐signaling components, mRNA levels of TRIB3 were significantly increased 24 h following polytrauma. TRIB3 can inhibit AKT kinase activity and increased TRIB3 gene expression is associated with systemic insulin resistance in humans (Oberkofler et al. 2010). Furthermore, skeletal muscle TRIB3 increases in streptozotocin‐treated, db/db mice and in Zucker fatty rats, and is correlated with decreased AKT signaling (Liu et al. 2010). Our data suggests that increased TRIB3 mRNA may indicate a novel role for TRIB3 in acute injury‐induced insulin resistance, which merits further investigation.

**Figure 6:**
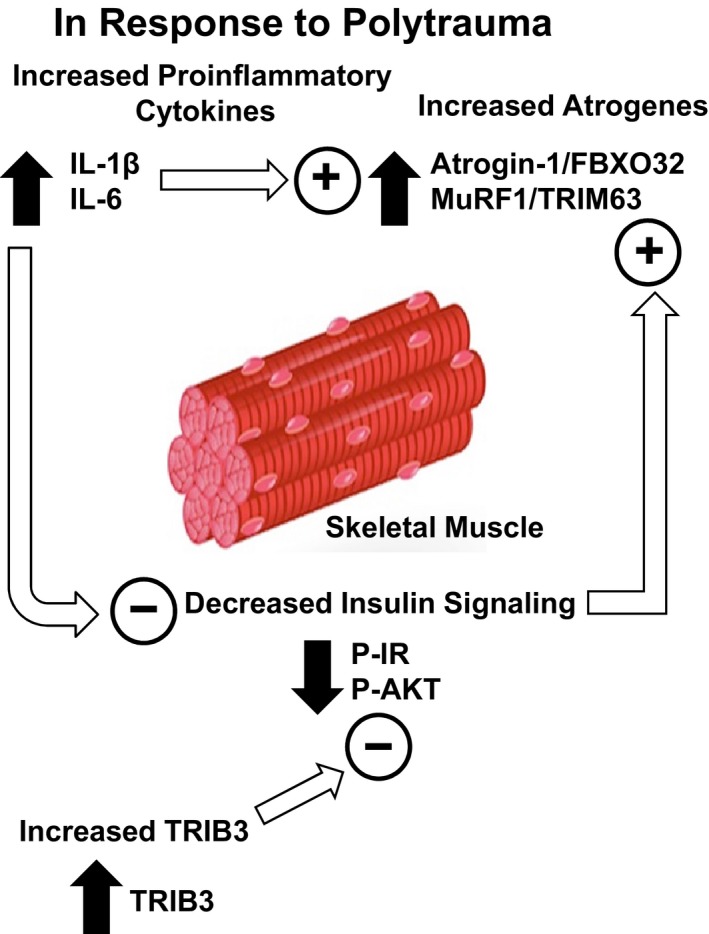
Summary of the effects of polytrauma on skeletal muscle. Proinflammatory cytokines (IL‐1β and IL‐6) are increased following polytrauma. These increases likely contribute to decreased insulin‐inducible signaling in skeletal muscle as measured by decreased phosphorylation of the insulin receptor and AKT. Increased TRIB3 may also contribute to the decrease in insulin signaling. Increased proinflammatory cytokines and decreased insulin signaling both may have a role in increased expression of atrogenes (Atrogin‐1/FBXO32 and MuRF‐1/TRIM63). The solid arrows are based on the data obtained in this study. The open arrows and the circled + and − signs are the interactions of these different pathways following polytrauma based on previously published studies.

In conclusion, the current studies demonstrated injury and time‐dependent increases in proinflammatory cytokine mRNA levels in skeletal muscle. These increases in proinflammatory cytokine may influence increased ubiquitin E3 ligase mRNA levels and insulin resistance following polytrauma (Fig. 6). Changes in IR and IRS‐1 protein levels following both single injuries and polytrauma were observed. However, insulin resistance, as measured by downstream signaling, was only observed 24 h following polytrauma. This data suggests alternative, polytrauma‐specific mechanisms are causative in skeletal muscle insulin resistance. This study provides evidence for a potentially novel role for TRIB3 in injury‐induced skeletal muscle insulin resistance (Fig. 6). This data demonstrates a number of pathological measures in skeletal muscle that is remote from the site of injury, triceps not being directly affected by a burn to the dorsum or abdominal injury by CLP. The deleterious effects of polytrauma in skeletal muscle likely contribute to complex systemic sequelae of polytrauma. Understanding the effects of polytrauma on skeletal muscle (Fig. 6) may provide insight into the numerous systemic pathologies, including metabolic derangement and an exacerbated immune response, following multiple, simultaneous injuries and may suggest improved treatment strategies.

## Conflict of Interest

None declared.

## References

[phy212659-bib-0001] Atiyeh, B. S. , S. W. Gunn , and S. N. Hayek . 2007 Military and civilian burn injuries during armed conflicts. Ann. Burns Fire Disasters 20:203–215. 21991098 PMC3188083

[phy212659-bib-0002] Barreiro, G. C. , R. R. Prattali , C. T. Caliseo , F. Y. Fugiwara , M. Ueno , P. O. Prada , et al. 2004 Aspirin inhibits serine phosphorylation of IRS‐1 in muscle and adipose tissue of septic rats. Biochem. Biophys. Res. Commun. 320:992–997. 15240146 10.1016/j.bbrc.2004.06.048

[phy212659-bib-0003] Bodine, S. C. , E. Latres , S. Baumhueter , V. K. Lai , L. Nunez , B. A. Clarke , et al. 2001 Identification of ubiquitin ligases required for skeletal muscle atrophy. Science 294:1704–1708. 11679633 10.1126/science.1065874

[phy212659-bib-0004] Bonaldo, P. , and M. Sandri . 2013 Cellular and molecular mechanisms of muscle atrophy. Dis. Model Mech. 6:25–39. 23268536 10.1242/dmm.010389 PMC3529336

[phy212659-bib-0005] Bortoff, K. D. , A. B. Keeton , J. L. Franklin , and J. L. Messina . 2010 Anti‐inflammatory action of insulin via induction of Gadd45‐beta transcription by the mTOR signaling pathway. Hepat. Med. 2:79–85. 21286247 10.2147/HMER.S7083 PMC3030126

[phy212659-bib-0006] Butcher, N. , and Z. J. Balogh . 2009 The definition of polytrauma: the need for international consensus. Injury 40(Suppl. 4):S12–S22. 19895948 10.1016/j.injury.2009.10.032

[phy212659-bib-0007] Calisto, K. L. , A. C. Camacho , F. C. Mittestainer , B. M. Carvalho , D. Guadagnini , J. B. Carvalheira , et al. 2012 Diacerhein attenuates the inflammatory response and improves survival in a model of severe sepsis. Crit. Care 16:R158. 22897821 10.1186/cc11478 PMC3580748

[phy212659-bib-0008] Castillero, E. , N. Alamdari , Z. Aversa , A. Gurav , and P. O. Hasselgren . 2013 PPARbeta/delta regulates glucocorticoid‐ and sepsis‐induced FOXO1 activation and muscle wasting. PLoS ONE 8:e59726. 23555761 10.1371/journal.pone.0059726 PMC3605288

[phy212659-bib-0009] Claassen, L. , S. Papst , K. Reimers , C. Stukenborg‐Colsman , L. Steinstraesser , P. M. Vogt , et al. 2014 Inflammatory response to burn trauma: nicotine attenuates proinflammatory cytokine levels. Eplasty 14:e46. 25671045 PMC4276106

[phy212659-bib-0010] Diao, L. , A. H. Marshall , X. Dai , E. Bogdanovic , A. Abdullahi , S. Mini‐Nik , et al. 2014 Burn plus lipopolysaccharide augments endoplasmic reticulum stress and NLRP3 inflammasome activation and reduces PGC‐1alpha in liver. Shock 41:138–144. 24434416 10.1097/SHK.0000000000000075 PMC4635464

[phy212659-bib-0011] Dismounted Complex Blast Injury Task Force, Caravalho, J. Jr, US Army Chair, DCBI Task Force . 2011 Dismounted complex blast injury: report of the army dismounted complex blast injury task force. 1–47.

[phy212659-bib-0012] Frost, R. A. , G. J. Nystrom , L. S. Jefferson , and C. H. Lang . 2007 Hormone, cytokine, and nutritional regulation of sepsis‐induced increases in atrogin‐1 and MuRF1 in skeletal muscle. Am. J. Physiol. Endocrinol. Metab. 292:E501–E512. 17003238 10.1152/ajpendo.00359.2006

[phy212659-bib-0013] Gilpin, D. A. 1996 Calculation of a new Meeh constant and experimental determination of burn size. Burns 22:607–611. 8982538 10.1016/s0305-4179(96)00064-2

[phy212659-bib-0014] Hansen, T. K. , S. Thiel , P. J. Wouters , J. S. Christiansen , and G. Van den Berghe . 2003 Intensive insulin therapy exerts antiinflammatory effects in critically ill patients and counteracts the adverse effect of low mannose‐binding lectin levels. J. Clin. Endocrinol. Metab. 88:1082–1088. 12629088 10.1210/jc.2002-021478

[phy212659-bib-0015] Herndon, D. N. , D. W. Wilmore , and A. D. Mason Jr . 1978 Development and analysis of a small animal model simulating the human postburn hypermetabolic response. J. Surg. Res. 25:394–403. 713539 10.1016/s0022-4804(78)80003-1

[phy212659-bib-0016] Jeschke, M. G. , D. Klein , and D. N. Herndon . 2004 Insulin treatment improves the systemic inflammatory reaction to severe trauma. Ann. Surg. 239:553–560. 15024317 10.1097/01.sla.0000118569.10289.ad PMC1356261

[phy212659-bib-0017] Keel, M. , and O. Trentz . 2005 Pathophysiology of polytrauma. Injury 36:691–709. 15910820 10.1016/j.injury.2004.12.037

[phy212659-bib-0018] Lang, C. H. , and R. A. Frost . 2007 Sepsis‐induced suppression of skeletal muscle translation initiation mediated by tumor necrosis factor alpha. Metabolism 56:49–57. 17161226 10.1016/j.metabol.2006.08.025

[phy212659-bib-0019] Lang, C. H. , D. Huber , and R. A. Frost . 2007 Burn‐induced increase in atrogin‐1 and MuRF‐1 in skeletal muscle is glucocorticoid independent but downregulated by IGF‐I. Am. J. Physiol. Regul. Integr. Comp. Physiol. 292:R328–R336. 16946078 10.1152/ajpregu.00561.2006

[phy212659-bib-0020] Lecky, F. E. , O. Bouamra , M. Woodford , R. Alexandrescu , and S. J. O'Brien . 2010 Epidemiology of polytrauma Pp. 13–25 in Pape H. C., Peitzman A. B., Schwab C. W. and Giannoudis P. V., eds. Damage control management in the polytrauma patient. Springer, New York.

[phy212659-bib-0021] Leffler, M. , T. Hrach , M. Stuerzl , R. E. Horch , D. N. Herndon , and M. G. Jeschke . 2007 Insulin attenuates apoptosis and exerts anti‐inflammatory effects in endotoxemic human macrophages. J. Surg. Res. 143:398–406. 17583747 10.1016/j.jss.2007.01.030

[phy212659-bib-0022] Li, Y. P. , Y. Chen , J. John , J. Moylan , B. Jin , D. L. Mann , et al. 2005 TNF‐alpha acts via p38 MAPK to stimulate expression of the ubiquitin ligase atrogin1/MAFbx in skeletal muscle. FASEB J. 19:362–370. 15746179 10.1096/fj.04-2364com PMC3099533

[phy212659-bib-0023] Li, L. , L. H. Thompson , L. Zhao , and J. L. Messina . 2009a Tissue specific difference in the molecular mechanisms for the development of acute insulin resistance following injury. Endocrinology 150:24–32. 18801909 10.1210/en.2008-0742 PMC2630903

[phy212659-bib-0024] Li, W. , J. S. Moylan , M. A. Chambers , J. Smith , and M. B. Reid . 2009b Interleukin‐1 stimulates catabolism in C2C12 myotubes. Am. J. Physiol. Cell Physiol. 297:C706–C714. 19625606 10.1152/ajpcell.00626.2008 PMC2740393

[phy212659-bib-0025] Liu, J. , X. Wu , J. L. Franklin , J. L. Messina , H. S. Hill , D. R. Moellering , et al. 2010 Mammalian Tribbles homolog 3 impairs insulin action in skeletal muscle: role in glucose‐induced insulin resistance. Am. J. Physiol. Endocrinol. Metab. 298:E565–E576. 19996382 10.1152/ajpendo.00467.2009 PMC2838520

[phy212659-bib-0026] Lu, X. M. , R. Tompkins , and A. Fischman . 2013 Burn injury‐induced IRS‐1 degradation in mouse skeletal muscle. Int. J. Burns Trauma 3:37–48. 23386984 PMC3560490

[phy212659-bib-0027] Ma, L. , C. Shen , J. Chai , H. Yin , H. Deng , and R. Feng . 2015 Extracellular signal‐regulated kinase‐mammalian target of rapamycin signaling and forkhead‐box transcription factor 3a phosphorylation are involved in testosterone's effect on severe burn injury in a rat model. Shock 43:85–91. 25057926 10.1097/SHK.0000000000000244

[phy212659-bib-0028] Matos, A. , E. R. Ropelle , J. R. Pauli , M. J. Frederico , R. A. de Pinho , L. A. Velloso , et al. 2010 Acute exercise reverses TRB3 expression in the skeletal muscle and ameliorates whole body insulin sensitivity in diabetic mice. Acta. Physiol. (Oxf) 198:61–69. 19681769 10.1111/j.1748-1716.2009.02031.x

[phy212659-bib-0029] Matsuda, N. , S. Yamamoto , H. Yokoo , K. Tobe , and Y. Hattori . 2009 Nuclear factor‐kappaB decoy oligodeoxynucleotides ameliorate impaired glucose tolerance and insulin resistance in mice with cecal ligation and puncture‐induced sepsis. Crit. Care Med. 37:2791–2799. 19707125 10.1097/CCM.0b013e3181ab844d

[phy212659-bib-0030] Mattick, J. S. , Q. Yang , M. A. Orman , M. G. Ierapetritou , F. Berthiaume , S. C. Gale , et al. 2013 Impact of burn priming on immune and metabolic functions of whole liver in a rat cecal ligation and puncture model. Int. J. Burns Trauma 3:55–65. 23386986 PMC3560487

[phy212659-bib-0031] Merritt, E. K. , A. Thalacker‐Mercer , J. M. Cross , S. T. Windham , S. J. Thomas , and M. M. Bamman . 2013 Increased expression of atrogenes and TWEAK family members after severe burn injury in nonburned human skeletal muscle. J. Burn. Care Res. 34:e297–e304. 23816995 10.1097/BCR.0b013e31827a2a9c PMC3770758

[phy212659-bib-0032] Nakazawa, H. , M. Yamada , T. Tanaka , J. Kramer , Y. M. Yu , A. J. Fischman , et al. 2015 Role of protein farnesylation in burn‐induced metabolic derangements and insulin resistance in mouse skeletal muscle. PLoS ONE 10:e0116633. 25594415 10.1371/journal.pone.0116633 PMC4296934

[phy212659-bib-0033] Nardi, G. M. , K. Scheschowitsch , D. Ammar , S. K. de Oliveira , T. B. Arruda , and J. Assreuy . 2014 Neuronal nitric oxide synthase and its interaction with soluble guanylate cyclase is a key factor for the vascular dysfunction of experimental sepsis. Crit. Care Med. 42:e391–e400. 24717470 10.1097/CCM.0000000000000301

[phy212659-bib-0034] Neunaber, C. , C. Zeckey , H. Andruszkow , M. Frink , P. Mommsen , C. Krettek , et al. 2011 Immunomodulation in polytrauma and polymicrobial sepsis ‐ where do we stand? Recent Pat. Inflamm. Allergy Drug Discov. 5:17–25. 21158733 10.2174/187221311794474892

[phy212659-bib-0035] Nunes, A. L. , J. B. Carvalheira , C. R. Carvalho , S. L. Brenelli , and M. J. Saad . 2001 Tissue‐specific regulation of early steps in insulin action in septic rats. Life Sci. 69:2103–2112. 11669454 10.1016/s0024-3205(01)01288-7

[phy212659-bib-0036] Oberkofler, H. , A. Pfeifenberger , S. Soyal , T. Felder , P. Hahne , K. Miller , et al. 2010 Aberrant hepatic TRIB3 gene expression in insulin‐resistant obese humans. Diabetologia 53:1971–1975. 20461355 10.1007/s00125-010-1772-2

[phy212659-bib-0037] Orman, M. A. , M. G. Ierapetritou , F. Berthiaume , and I. P. Androulakis . 2012 Long‐term dynamic profiling of inflammatory mediators in double‐hit burn and sepsis animal models. Cytokine 58:307–315. 22402033 10.1016/j.cyto.2012.01.017 PMC3355995

[phy212659-bib-0038] Owens, B. D. , J. F. Kragh Jr , J. C. Wenke , J. Macaitis , C. E. Wade , and J. B. Holcomb . 2008 Combat wounds in operation Iraqi Freedom and operation Enduring Freedom. J. Trauma 64:295–299. 18301189 10.1097/TA.0b013e318163b875

[phy212659-bib-0039] Peng, Z. Y. , J. V. Bishop , X. Y. Wen , M. M. Elder , F. Zhou , A. Chuasuwan , et al. 2014 Modulation of chemokine gradients by apheresis redirects leukocyte trafficking to different compartments during sepsis, studies in a rat model. Crit. Care 18:R141. 24992991 10.1186/cc13969 PMC4227131

[phy212659-bib-0040] Pfeifer, R. , I. S. Tarkin , B. Rocos , and H. C. Pape . 2009 Patterns of mortality and causes of death in polytrauma patients–has anything changed? Injury 40:907–911. 19540488 10.1016/j.injury.2009.05.006

[phy212659-bib-0041] Pidcoke, H. F. , L. A. Baer , X. Wu , S. E. Wolf , J. K. Aden , and C. E. Wade . 2014 Insulin effects on glucose tolerance, hypermetabolic response, and circadian‐metabolic protein expression in a rat burn and disuse model. Am. J. Physiol. Regul. Integr. Comp. Physiol. 307:R1–R10. 24760998 10.1152/ajpregu.00312.2013 PMC4080276

[phy212659-bib-0042] Quintana, H. T. , J. A. Bortolin , N. T. da Silva , F. A. Ribeiro , E. A. Liberti , D. A. Ribeiro , et al. 2015 Temporal study following burn injury in young rats is associated with skeletal muscle atrophy, inflammation and altered myogenic regulatory factors. Inflamm. Res. 64:53–62. 25413930 10.1007/s00011-014-0783-8

[phy212659-bib-0043] Ramasamy, A. , A. M. Hill , and J. C. Clasper . 2009 Improvised explosive devices: pathophysiology, injury profiles and current medical management. J. R. Army Med. Corps 155:265–272. 20397601 10.1136/jramc-155-04-05

[phy212659-bib-0044] Ramasamy, A. , S. D. Masouros , N. Newell , A. M. Hill , W. G. Proud , K. A. Brown , et al. 2011 In‐vehicle extremity injuries from improvised explosive devices: current and future foci. Philos. Trans. R. Soc. Lond. B Biol. Sci. 366:160–170. 21149353 10.1098/rstb.2010.0219 PMC3013426

[phy212659-bib-0045] Santaniello, J. M. , F. A. Luchette , T. J. Esposito , H. Gunawan , R. L. Reed , K. A. Davis , et al. 2004 Ten year experience of burn, trauma, and combined burn/trauma injuries comparing outcomes. J. Trauma 57:696–700. 15514521 10.1097/01.ta.0000140480.50079.a8

[phy212659-bib-0046] Shere‐Wolfe, R. F. , S. M. Galvagno Jr , and T. E. Grissom . 2012 Critical care considerations in the management of the trauma patient following initial resuscitation. Scand J. Trauma Resusc. Emerg. Med. 20:68. 22989116 10.1186/1757-7241-20-68 PMC3566961

[phy212659-bib-0047] Sheriff, S. , R. Joshi , L. A. Friend , J. H. James , and A. Balasubramaniam . 2009 Ghrelin receptor agonist, GHRP‐2, attenuates burn injury‐induced MuRF‐1 and MAFbx expression and muscle proteolysis in rats. Peptides 30:1909–1913. 19577604 10.1016/j.peptides.2009.06.029

[phy212659-bib-0048] Sritharan, K. , and H. Thompson . 2009 Understanding the metabolic response to trauma. Br. J. Hosp. Med. (Lond) 70:M156–M158. 19966719 10.12968/hmed.2009.70.Sup10.44641

[phy212659-bib-0049] Steiner, J. L. , and C. H. Lang . 2015 Sepsis attenuates the anabolic response to skeletal muscle contraction. Shock 43:344–351. 25423127 10.1097/SHK.0000000000000304 PMC4359659

[phy212659-bib-0050] Sugita, H. , M. Kaneki , M. Sugita , T. Yasukawa , S. Yasuhara , and J. A. Martyn . 2005 Burn injury impairs insulin‐stimulated Akt/PKB activation in skeletal muscle. Am. J. Physiol. Endocrinol. Metab. 288:E585–E591. 15536206 10.1152/ajpendo.00321.2004

[phy212659-bib-0051] Tang, H. , K. Inoki , M. Lee , E. Wright , A. Khuong , A. Khuong , et al. 2014 mTORC1 promotes denervation‐induced muscle atrophy through a mechanism involving the activation of FoxO and E3 ubiquitin ligases. Sci. Signal. 7:ra18. 24570486 10.1126/scisignal.2004809

[phy212659-bib-0052] Thompson, L. H. , H. T. Kim , Y. Ma , N. A. Kokorina , and J. L. Messina . 2008 Acute, muscle‐type specific insulin resistance following injury. Mol. Med. 14:715–723. 19009015 10.2119/2008-00081.Thompson PMC2582859

[phy212659-bib-0053] Tsao, C. M. , K. Y. Li , S. J. Chen , S. M. Ka , W. J. Liaw , H. C. Huang , et al. 2014 Levosimendan attenuates multiple organ injury and improves survival in peritonitis‐induced septic shock: studies in a rat model. Crit. Care 18:652. 25432865 10.1186/s13054-014-0652-4 PMC4274679

[phy212659-bib-0054] Tsujinaka, T. , J. Fujita , C. Ebisui , M. Yano , E. Kominami , K. Suzuki , et al. 1996 Interleukin 6 receptor antibody inhibits muscle atrophy and modulates proteolytic systems in interleukin 6 transgenic mice. J. Clin. Invest. 97:244–249. 8550842 10.1172/JCI118398 PMC507086

[phy212659-bib-0055] Wichterman, K. A. , A. E. Baue , and I. H. Chaudry . 1980 Sepsis and septic shock–a review of laboratory models and a proposal. J. Surg. Res. 29:189–201. 6997619 10.1016/0022-4804(80)90037-2

[phy212659-bib-0056] Xu, J. , S. Ji , D. Y. Venable , J. L. Franklin , and J. L. Messina . 2005 Prolonged insulin treatment inhibits GH signaling via STAT3 and STAT1. J. Endocrinol. 184:481–492. 15749807 10.1677/joe.1.05977

[phy212659-bib-0057] Xu, J. , H. T. Kim , Y. Ma , L. Zhao , L. Zhai , N. Kokorina , et al. 2008 Trauma and hemorrhage‐induced acute hepatic insulin resistance: dominant role of tumor necrosis factor (TNF)‐alpha. Endocrinology 149:2369–2382. 18187553 10.1210/en.2007-0922 PMC2329283

[phy212659-bib-0058] Yuan, L. , J. Han , Q. Meng , Q. Xi , Q. Zhuang , Y. Jiang , et al. 2015 Muscle‐specific E3 ubiquitin ligases are involved in muscle atrophy of cancer cachexia: an in vitro and in vivo study. Oncol. Rep. 33:2261–2268. 25760630 10.3892/or.2015.3845

[phy212659-bib-0059] Zhai, L. , and J. L. Messina . 2009 Age and tissue specific differences in the development of acute insulin resistance following injury. J. Endocrinol. 203:365–374. 19752148 10.1677/JOE-09-0269 PMC2929648

[phy212659-bib-0060] Zhang, Q. , E. A. Carter , B. Y. Ma , M. White , A. J. Fischman , and R. G. Tompkins . 2005 Molecular mechanism(s) of burn‐induced insulin resistance in murine skeletal muscle: role of IRS phosphorylation. Life Sci. 77:3068–3077. 15982669 10.1016/j.lfs.2005.02.034

